# VR-guided exercise and mindfulness program for people with chronic pain: a randomised controlled cross-over pilot trial

**DOI:** 10.1186/s13102-025-01102-9

**Published:** 2025-03-21

**Authors:** Sella Aarrestad Provan, Giovanna Calogiuri, Linda Røset, Maren Mariussen, Ingeborg Rosøy, Tonje Jossie Johnsen, Thomas Johansen, Ole Einar Flaten, Sigbjørn Litleskare

**Affiliations:** 1 Department of Public Health and Sport Sciences, Faculty of Social and Health Sciences, Inland Norway University of Applied Sciences, Elverum, Norway; 2 Department of Rheumatology, Center for Treatment of Rheumatic and Musculoskeletal Diseases (REMEDY), Diakonhjemmet Hospital, Oslo, Norway; 3 Department of Nursing and Health Sciences, Centre for Health and Technology, University of South- Eastern Norway, Drammen, Norway; 4 Hernes Occupational Rehabilitation Centre, Instituttvegen 34, Hernes, 2410 Norway; 5 Norwegian National Advisory Unit on Occupational Rehabilitation, Haddlandsvegen 20, Rauland, 3864 Norway; 6 Game School-Department of Game Development, Inland Norway University of Applied Sciences, Elverum, Norway

**Keywords:** Rehabilitation, Chronic pain, Exercise, Mindfulness, VR

## Abstract

**Background:**

Physical exercises and mindfulness are important components in the management of chronic pain, but pain may reduce exercise adherence. Virtual reality (VR) can provide cognitive inhibition of the ascending pain signal and may thus be a tool for the delivery of pain management during exercise interventions. In this study we assessed a VR-guided intervention seeking to improve physical fitness in individuals with chronic pain.

**Methods:**

Participants in rehabilitation for chronic pain were included in a randomised controlled pilot trial with a cross-over design. In counter-balanced order participants were asked to perform, five minutes of aerobic exercise following identical instructions given through either a VR headset or television (TV) screen. The procedures were then repeated with mindfulness exercises. Heart rate (HR) was monitored throughout all four sessions and participants self-reported perceived exercise intensity, benefit, relaxation, and reward. Paired Student’s t-test, Wilcoxon signed rank test and McNemar’s test were performed to compare the outcome variables across sessions for individuals, as appropriate. (Clinical trial registration NCT06611566 09.09.24, retrospectively registered).

**Results:**

Twenty-seven participants were included in the study. The mean age (SD) was 40.4 (11.3) years, and 17 (63%) were men. Mean HR, the proportion of time spent at moderate-vigorous exercise intensity levels, and all self-reported measurements were comparable between the VR vs. TV sessions. No major adverse events were reported. The physiological and perceived exercise outputs of aerobic exercises were thus similar across modes of delivery (VR vs. TV) in individuals with chronic pain.

**Conclusions:**

This study confirms the possibilities of VR-guided interventions in the pain management of individuals with chronic pain with comparable levels of exertion to TV-guided exercise and few adverse events. The promise of VR-guided mindfulness in the rehabilitation of patients with chronic pain conditions is also confirmed.

**Supplementary Information:**

The online version contains supplementary material available at 10.1186/s13102-025-01102-9.

## Introduction

Chronic widespread pain (CWP) is a condition where central pain-sensitization is considered an important part of the pathology [1]. CWP is more frequent in women than in men, and the prevalence in Norway has been reported to be between 3.2 and 10% [2]. CWP is a common symptom among individuals with fibromyalgia, in addition to fatigue, sleep disturbance, anxiety and depression [1]. The management of CWP can be frustrating as there is no “quick fix”, with limited evidence for treatment modalities and no available efficacious medication. There is, however, a consensus that a combination of physical and cognitive interventions is key in the management of symptoms [3].

### Physical activity and mindfulness in the management of chronic pain

Regular physical exercise is vital for health and reduces the risk of cardiovascular events and early mortality [4]. In order to improve physical fitness, the American College of Sports Medicine (ACSM) recommends moderate-intensity cardiorespiratory exercise training for at least 150 min per week or vigorous-intensity exercise for at least 75 min per week or equivalent combinations of moderate- and vigorous-intensity exercise, to the general population [4]. The heart rate (HR) during the exercise is a measure of exercise intensity and adherence to the exercise protocol [4]. According to two recent systematic reviews individuals with fibromyalgia have significantly reduced physical fitness compared to matched healthy controls [5] and the majority do not attain the recommended levels of physical activity [6]. The low adherence to physical activity is unfortunate because physical activity of either high and low intensity has been found to have positive impact on pain, depression, and fatigue [7].

Effective pain management is crucial in connection with physical activity, as pain prior to and during exercise is a major barrier to physical activity in individuals with CWP [6, 8]. The drop-out rates for exercise interventions are lower in exergames, (defined as the playing of video games that require physical exercise and are intended as a work-out [9]), and in supervised programs and this should be kept in mind when developing novel programs [10].

Mindfulness is defined as awareness of one’s internal states and surroundings (https://www.apa.org/topics/mindfulness). Two systematic reviews have summarised the evidence for mindfulness on chronic pain and fibromyalgia. They find small to moderate effect sizes for pain, depression, anxiety and quality of life, compared to waitlist, treatment as usual or education [11, 12]. Mindfulness is thus recommended as an adjunctive treatment in CWP, and a core-treatment in individuals who have depression as an additional symptom [3, 13].

### Virtual reality: Potential and challenges

Virtual reality (VR), generally delivered though head-mounted displays (HMD), has become increasingly popular over the past decade. Studies indicate that during VR experiences, the patient’s attention is drawn away from the bodily pain signals, providing a cognitive inhibition of the ascending pain signal and in some cases reducing the perceived pain intensity [14, 15]. A limitation of these studies is that the VR intervention was administered *in addition* to the regular treatment, hence leaving the question of whether VR may increase the tolerance to physical and/or cognitive exercises largely unanswered. The experience of a VR-delivered exercise intervention has also not been compared to other forms of remotely delivery exercise, such as a program delivered on television (TV)-screen. Importantly, no study has measured heart rate (HR) while performing VR-guided exercises as an indicator of adherence to the exercise protocol and level of intensity. Moreover, the extent of literature in this field is yet limited, and researchers have called for studies testing such tailored interventions [16].

The objectives of this study were thus to assess whether physical exercise and mindfulness interventions can be effectively delivered through a VR headset in individuals with chronic pain, compared to non-immersive delivery, by:

1. comparing HR, self-reported adherence and exercise intensity in individuals performing the same aerobic exercise session guided through VR vs. a TV-screen.
2. comparing the HR, perceived relaxation and reward in individuals performing the same mindfulness session guided through VR vs. a TV-screen.
3. recording the occurrence of adverse events during any of the VR-guided sessions.

## Methods

A VR-guided exercise and mindfulness program (VRalgia), alongside the protocol of the pilot study presented in this paper, were designed through a participatory process described elsewhere [17]. Participants were recruited through partner patients’ organizations, a local exercise group engaging people living with rheumatological conditions, and an inpatient occupations rehabilitation centre. The inclusion criteria were adults (> 18 years of age) with chronic musculoskeletal pain. The exclusion criterion was an inability to perform VR-delivered physical exercises. The study was conducted according to the Helsinki declaration and approved by the Local committee for medical and health research ethics at the Inland Norway University of Applied Sciences (Ref. 20616405). Informed consent was obtained from all patients. The study was retrospectively registered in clinicaltrials.gov 09.09.24 (NCT06611566).

### Study design and intervention

The study was designed as a randomised controlled trial with a cross-over design. Each participant underwent five exercise sessions in total. Firstly, all participants performed a six- minutes warm-up session following instructions projected on a TV screen. Subsequently, the participants performed two aerobic exercise sessions, identical in content and duration (five- minutes), once following the instructions from VR (VR-A) and once following the identical program displayed on a TV screen (TV-A). The allocation of the first aerobic exercise session as VR-A or TV-A was randomly assigned for each individual by the data-collectors through alternating the order of examination on alternate days. After completing the first aerobic exercise session, the participants were subsequently asked to complete the second session guided through the alternative mode of delivery. Each session was of five-minute duration in order to limit the burden to the patient who thus performed aerobic exercises for a total of 10-minutes. According to the ACSM, bouts of exercise of < 10-minutes may improve fitness levels [4].

After the exercise sessions the same randomised cross-over design was followed for two five- minute mindfulness sessions, with the participants undergoing, in a counter-balanced and random order, a guided mindfulness program, once delivered through VR (VR-M) and once displayed on a TV screen (TV-M). Please see supplementary Fig. 1 for an overview of the study design.

### Virtual environment and technology

In the VR-A and VR-M, the participants wore a stand-alone HMD (Oculus Quest 2 with stock head strap, Meta Platforms, Menlo Park, California, USA). The virtual environment was developed by Fynd Reality AS (Hamar, Norway), in line with general criteria outlined during the participatory process and consisted of a computer-generated replication of the town square of Hamar (Norway). Each participant would enter this town square as a virtual avatar with virtual arms and body. The position of the arms was tracked by the hand-held controllers. A large screen would appear in the virtual town square, showing a video in which an instructor guided an exercise session. The same video was shown on the TV screen.

### Data collection at baseline

All participants completed the Widespread Pain Index and Symptom Scale that together compose the Polysymptomatic Distress Scale (PDS) [18]. The PDS is composed of variables used in the 2010 American College of Rheumatology (ACR) fibromyalgia criteria which were later modified for use in clinical research and self-evaluation in epidemiological surveys [18–20]. The PDS is thus both a diagnostic aid and a measure of fibromyalgia severity [18]. A diagnosis of fibromyalgia may be indicated by a PDS score of ≥ 12 in an epidemiological study [1]. The PDS has been translated and validated in Norwegian [21].

### Outcome measures

HR was registered throughout the sessions using a HR-monitors worn at the wrist (Garmin^®^ Forerunner 55). The mean HRs for the last three minutes of each session were calculated for each individual and was the primary outcome.

This pilot study explored several secondary outcomes. For the aerobic exercise sessions, HR was also categorised into HR-zones according to the ACSM recommendations based on the participants’ predicted maximal HR (220 minus age) [4], with the proportion of time spent in “ moderate-vigorous exercise intensity” (i.e., HR > 65% of maximum heart rate) being calculated and used for further analysis.

Participants self-reported exercise intensity and personal adherence immediately after each aerobic session by completing a short paper questionnaire. The questions were phrased as follow: “How challenging was the session” and “How well did you exercise?” (Author’s translation). After each mindfulness sessions, the participants were asked to complete a short questionnaire regarding the degree of relaxation and perceived reward. The questions were phrased as follow: “How relaxing was the session?” and “How rewarding was the session?” All questions were rated on a 5-point Likert scale ranging from 1 (least imaginable) to 5 (most imaginable).

Adverse events were recorded directly after the session by asking the participants whether they experienced any accident, malaise, etc. during the VR sessions.

### Statistics

We compared the mean HR values recorded within the last three minutes of the warm-up vs. all other sessions, as well as between each aerobic exercise (VR-A vs. TV-A) and mindfulness sessions (VR-M vs. TV-M), using paired Student’s t-test.

The time spent in each HR category was compared using the paired Wilcoxon signed rank test and time in the “moderate-vigorous” vs. “low” ACSM zone in the VR-A vs. TV-A sessions using McNemar’s test for dichotomised variables.

Comparisons between the self-reported measurements collected after the aerobic exercise (VR-A vs. TV-A) and mindfulness sessions (VR-M vs. TV-M) were performed using the paired Wilcoxon signed rank test for each session for ordinal data and McNemar’s test for dichotomised variables.

This was a pilot study with a convenience sample and power analyses were therefore not performed.

## Results

Twenty-nine participants were included in the study, but due to technical difficulties (wifi connection malfunction at Hernes Institute on one occasion), only 27 performed the intervention. The mean age (SD) was 40.4 (11.3) years, and 17 (63%) were men. The median number (min-max) of self-reported painful sites were 6 [1–15] and 11 (41%) fulfilled the criteria for fibromyalgia according to the PDS [18]. There was a total of 20 103 recordings of heart rate from the last 4 min of each session, 2 440 during warm-up, 4273 during the VR-A, 4 345 during the TV-A, 4 087 during the VR-M and 4 366 during the TV-M sessions. No participants were lost to follow-up. Please see Table 1 for baseline characteristics.

**Table 1:** Baseline descriptives

Variables	Values
Age (mean years (min-max)))	40.4 (19–61)
Number of males (%)	17 (63)
**Self-reported health compared to peers**	**Numbers (%)**
- Very poor	9 (33.3)
- Poor	10 (37.0)
- Average	5 (18.5)
- Good	2 (7.4)
- Very good	1 (3.7)
**Self-reported fitnesss level compared to peers**	**Numbers (%)**
- Very poor	0
- Poor	10 (38.5)
- Average	11 (42.3)
- Good	5 (19.2)
- Very good	0
**Fibromyalgia survey diagnostic and severity scale**	**Score**
- Symptom scale	4.9 (3.4)
- Widespread pain index	6.2 (4.2)
Fibromyalgia criteria *n*(%)	11 (37.9)
Chronic widespread pain *n*(%)	12 (41.4)

Self-reported health and fitness level compared to peers given as a categorical variable

Fibromyaglia symptom scale score possible range 0–12

Fibromyalgia widespread pain index possible range 0–19

### Heart rate

The mean HR (SD) in the warm-up session was 98.5 (12.5) which was significantly lower than the mean HR in both aerobic sessions, *p* < 0.001, and significantly lower than the overall mean HR for both the mindfulness sessions, *p* < 0.001.

**Fig. 1:**
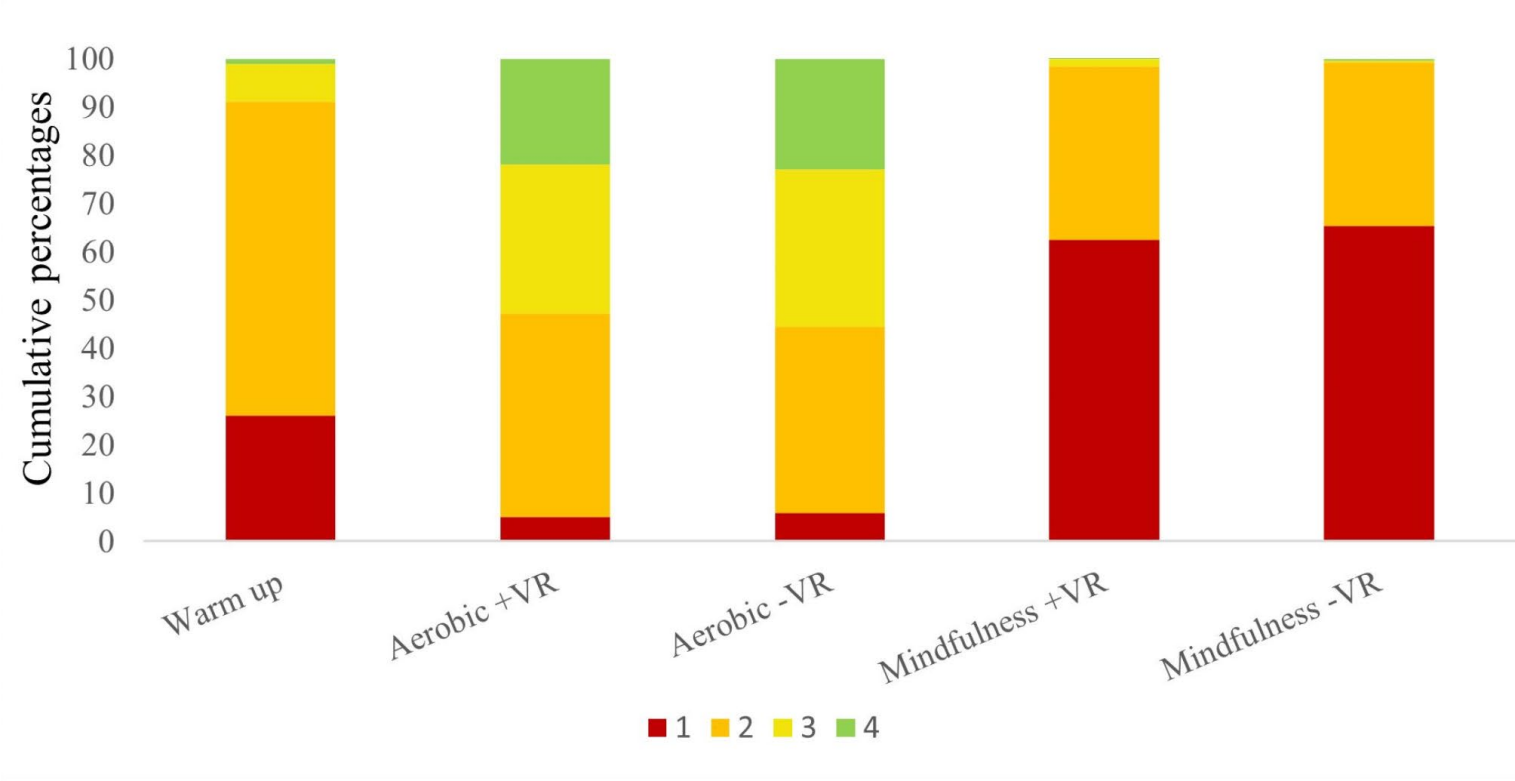
Distribution of time spent in each heart rate zone during VR-and TV-guided sessions. Heart-rate according to the American College of Sports Medicine recommendations based on the participants’ predicted maximal heart-rate (220 minus age). 1 = heart rate < 50% of maximal heart rate, 2 = heart rate ≥ 50% and < 65% of maximal heart, 3 = heart rate ≥ 65% and < 75% of maximal heart, 4 = heart rate ≥ 75% of maximal heart rate. VR; virtual reality TV; television

The mean HR in the VR-A session was 120.3 (18.7) vs. 119.8 (19.0) in the TV-A session, *p* = 0.57. Paired student’s t-test comparing HR during the aerobic sessions (VR-A vs. TV-A) was not statistically significant, *p* = 0.37. The proportion of HR recordings in the zone for moderate-vigorous exercise was 53.5% in the VR-A session, vs. 56.1% in the TV-A session McNemar’s exact *p* = 0.91 (Fig. 1).

The mean HR in the VR-M session was 83.9 (14.4) vs. 83.7 (13.4) in the TV-M session. *Paired Student’s t-test of heart rate in VR-M vs. TV-M did not find significant differences between groups*, *p* = 0.17 (Fig. 1).

### Self-reported measurements

**Fig. 2:**
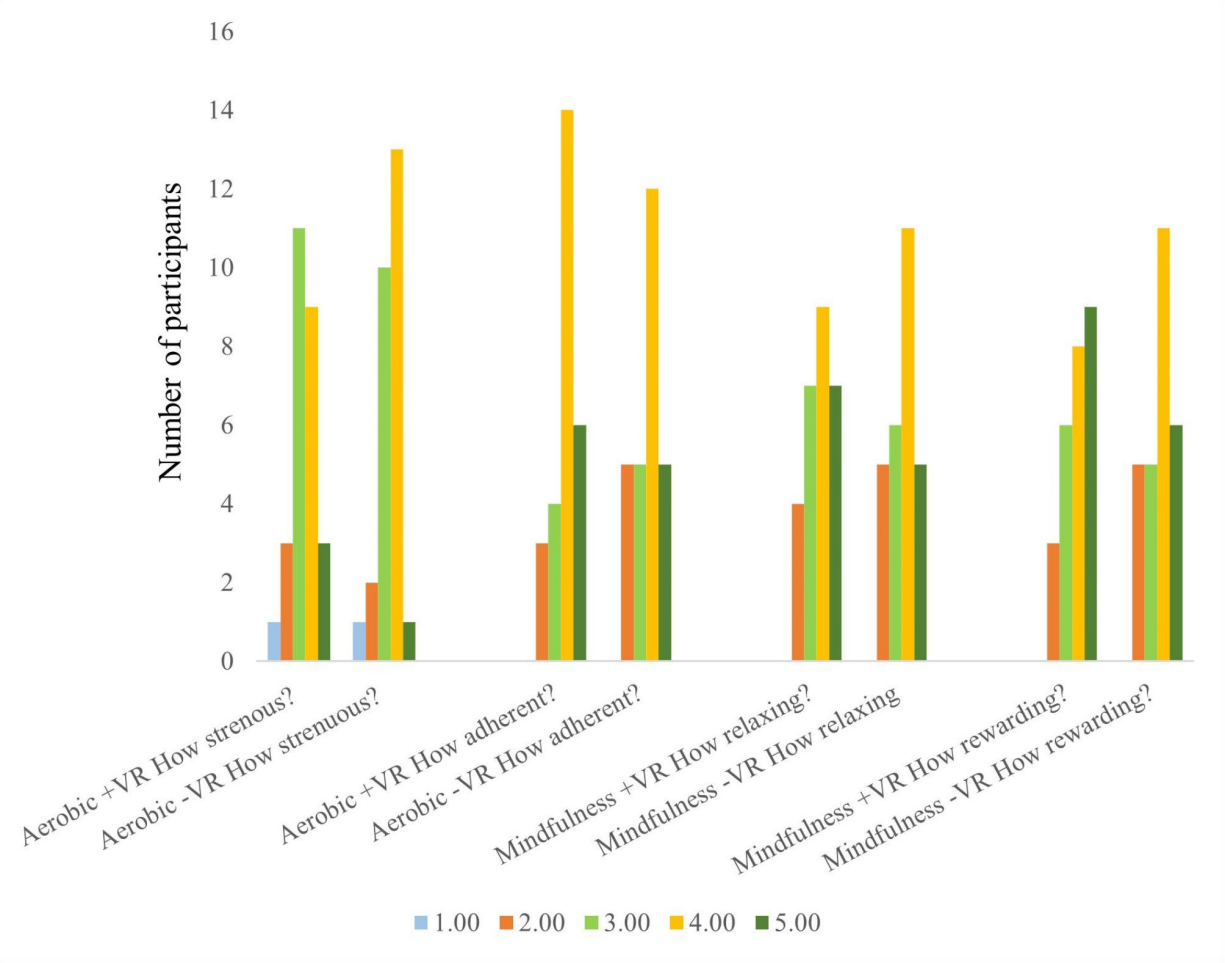
Self-reported outcomes following VR- and TV-guided sessions. VR; virtual reality, TV; television Numeric rating scale 1 = least, 5 = most

For the aerobic exercise sessions, 12 (44%) participants in the VR-A vs. 14 (52%) in the TV-A session reported that the degree of intensity was 4 or 5. The McNemar’s test found no statistical difference between these proportions (exact *p* = 0.69). The Wilcoxon signed rank test for paired ordinal data did not find a statistically significant difference between intensity in VR-A and TV-A (*p* = 0.58) (Fig. 2).

Regarding the exercise adherence, 20 (74%) of participants in the VR-A and 17 (63%) in the TV-A session reported a high degree of adherence (4 or 5 on a 5-point NRS)). The difference between sessions was not statistically significant, neither according to McNemar’s test (Exact *p* = 0.45) nor Wilcoxon signed rank test for paired ordinal data (*p* = 0.18). One person did not complete this question (Fig. 2).

For the mindfulness sessions, 16 participants (59%) in both VR-M and TV-M reported the degree of relaxation to be 4 or 5. Eleven (40%) participants did not give identical scores regarding relaxation in each modality, pointing to a variability regarding what modality was experienced as most relaxing. There were no statistically significant differences between dichotomous outcomes according to McNemar’s test (*p* = 1.0) or for the ordinal data according to the Wilcoxon signed rank test (exact *p* = 0.79) (Fig. 2).

For the perceived reward of the mindfulness session, 17 participants (63%) reported the perceived reward to be 4 or 5 in both the VR-M and the TV-M sessions. Nine participants did not report the exact same response in both sessions. There were no statistically significant differences in dichotomous outcomes according to McNemar’s test (*p* = 1.00). The Wilcoxon signed rank test for paired ordinal data was also not statistically significant (exact *p* = 0.40) (Fig. 2).

### Adverse events

There were no serious adverse events. Five participants reported becoming unbalanced, two reported dizziness and six participants felt that the VR exercise increased the level of musculoskeletal pain.

## Discussion

In individuals with CWP, a short aerobic exercise session delivered though VR resulted in an exercise intensity that was comparable, with regards to both HR and participants’ perceived intensity, to that of an identical session delivered through a TV screen. Further, in the same patient group a mindfulness intervention delivered though VR resulted in comparable HR and self-reported relaxation and reward, as a mindfulness intervention delivered through a TV screen.

Chronic widespread pain and fibromyalgia are musculoskeletal conditions that collectively exert a major impact on health care expenditures through treatment costs, disability payments and sick leave [2]. Identifying interventions that may be remotely delivered to both urban and rural communities, and with the potential of engaging participants over a lengthy treatment duration, is therefore of great importance.

A recently published scoping review identified nine papers which describe interventions of VR based exercise in individuals with chronic musculoskeletal pain, six of the papers were randomized controlled trials [22]. The interventions identified by the review ranged from core stabilizing to aerobic exercises, and all were delivered through fully immersive VR. A reduction in pain intensity was the outcome with the greatest level of evidence, and while there were also reports of improved functioning and reduced fear of movement, these studies only included individuals with chronic neck pain and the relevance to the current study is therefore limited. Interestingly, none of the studies measured HR while exercising and in general exercise intensity seems not to have been recorded, except for one study in which step-count was recorded while exercising [23]. Although the number of steps is a common indicator of moderate-intensity physical activity and often used for weight reduction purposes, exercise of moderate to vigorous intensity has been shown to be key to improving physical fitness [4]. In the current study we measured HR throughout the aerobic exercise sessions and estimated the time spent in vigorous exercise based on the percentage of maximum HR. This is in line with the ACSM recommendations and one of the most common methods for estimating exercise intensity [4]. The results showed similar HR responses during exercise guided by VR or TV screen, which suggests that VR-guided exercise does not impair exercise intensity. Thus, although this was not directly measured in our study, the use of VR may contribute to pain management during exercise in this patient group, although we have to acknowledge that 22% of participants reported an increased level of musculoskeletal pain during the VR-exercise session.

The evidence for VR-guided mindfulness has recently been synthesised in a scoping review by O’Connor et al. [24]. Interestingly VR guided mindfulness was found to be superior to audio-guided and sham-VR-guided mindfulness with respect to pain relief [25, 26]. The current study reports mean HR as well as degrees of relaxation and perceived reward that were similar in the VR- and TV-guided mindfulness sessions, although participants seemed to have different perceptions of which modality was most relaxing. We thus do not show a clear benefit of VR-guided mindfulness. Our findings are largely in line with previous studies, though the reporting of adverse events also highlights possible challenges as well as the benefit of different modes of delivery to match individual preferences. In this respect, a challenge commonly associated with VR is the possibility that some users experience cybersickness. This malaise, similar to motion-sickness, is believed to be caused by a sensory conflict among the visual, vestibular and proprioceptive inputs [27]. Cybersickness can have a dramatic impact on the effectiveness of a VR experience, as well as inducing negative emotional responses, resulting in increased stress and perceived fatigue even when a VR experience was designed to elicit relaxation [28]. Moreover, its symptoms can last long after the VR experience [29]. Hence, especially with vulnerable groups such as individuals with CWP, when planning VR-guided mindfulness interventions, it is important to consider the individual’s susceptibility and tolerance to cybersickness. It is thus reassuring that the adverse events reported regarding dizziness are in line with other studies and were mild [30]. Although we cannot preclude that dizziness may limit the clinical usefulness of VR-guided interventions, we believe that through continued use some participants may become increasingly accustomed to VR.

### Strengths and limitations

The current study was designed as a pilot trial and the participants were only exposed to one VR-guided exercise and mindfulness session. Adherence to the exercise protocol was assessed by heart rate which may have been confounded by pain or dizziness., Adherence may also have been confounded by the exertion of self-reporting between sessions and the fact that all sessions were performed during the course of one day. These issues, together with the short duration of the session and, are limitations of the study. The study was not powered to show statistical superiority. However, each participant acted as their own control, adding to the scientific validity of the study. Further, both interventions were delivered by screen and individually to participants thereby removing some of the bias that may have occurred if group exercises with inevitable social interactions were compared to VR-delivered interventions. The study design also allowed us to present identical sessions by VR and on TV-screen.

## Conclusion

Patients with CWP were able to attain similar levels of exercise intensity, assessed through objective and subjective indicators, during a structured aerobic exercises session irrespective of whether this was delivered through VR or a TV screen. Similarly, the individuals also experienced equivalent levels of HR, relaxation, and reward during a mindfulness exercises session, irrespective of mode of delivery. This confirms the possibilities of VR-guided interventions to improve physical fitness and induce relaxation in individuals with chronic pain.

## Electronic supplementary material

Below is the link to the electronic supplementary material.

Supplementary Material 1

Supplementary Material 2

## Data Availability

The datasets used and/or analyzed during the current study are available from the corresponding author on reasonable request.

## Abbreviations

ACSM: American College of Sports Medicine
CWP: Chronic widespread pain
HMD: Head-mounted display
HR: Heart rate
SD: Standard deviation
TV: Television
VR: Virtual reality

## Declarations

### CONSORT guidelines

The manuscript complies with the CONSORT guidelines and the checklist has been uploaded.
