# Supplementary figures and images for: VR-guided exercise and mindfulness program for people with chronic pain: a randomised controlled cross-over pilot trial
Source: BMC Sports Sci Med Rehabil. 2025 Mar 21;17:55. doi: 10.1186/s13102-025-01102-9 (PMC11927144; doi:10.1186/s13102-025-01102-9)

## Supplementary figure 1

### Overview of study design

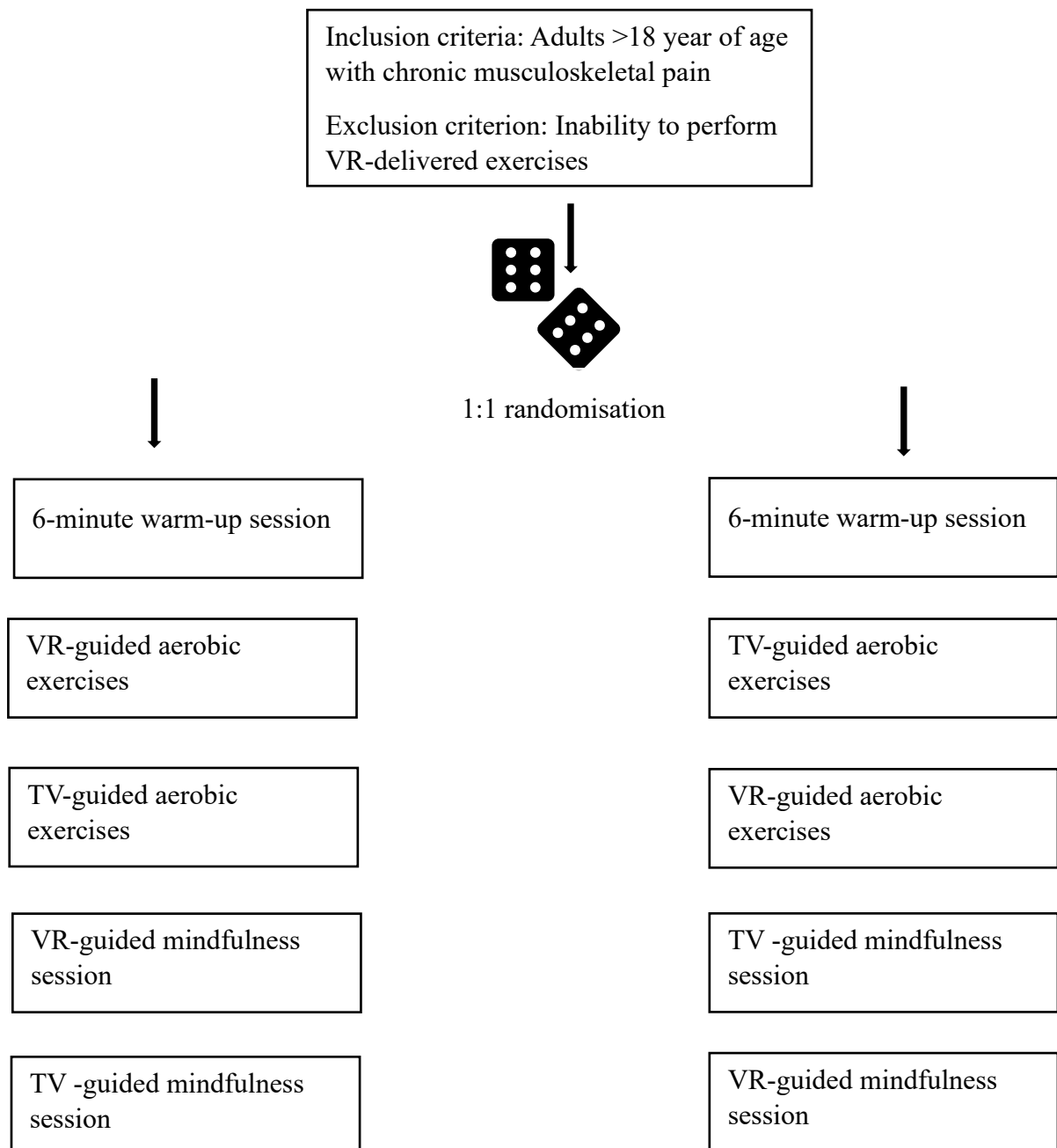

Footnote: VR; virtual reality TV; television

Supplement: Supplementary file 2 — Supplementary Material 2 [file 13102_2025_1102_MOESM2_ESM.pdf]
